# Comorbidities in heart failure with preserved ejection fraction

**DOI:** 10.1007/s00059-022-05123-9

**Published:** 2022-06-08

**Authors:** Andrea Deichl, Rolf Wachter, Frank Edelmann

**Affiliations:** 1 Medizinische Klinik mit Schwerpunkt Kardiologie, Charité—Universitätsmedizin Berlin, Campus Virchow-Klinikum (CVK), Augustenburger Platz 1, 13353 Berlin, Germany; 2 Standort Berlin, DZHK (Deutsches Zentrum für Herz-Kreislauf-Forschung), Berlin, Germany; 3 Klinik und Poliklinik für Kardiologie, Universitätsklinikum Leipzig, Leipzig, Germany; 4 Klinik für Kardiologie und Pneumologie, Universitätsmedizin Göttingen, Göttingen, Germany; 5 Standort Göttingen, DZHK (Deutsches Zentrum für Herz-Kreislauf-Forschung), Göttingen, Germany

**Keywords:** Congestive heart failure, HFpEF, Prevalence, Treatment options, Prognosis, Kongestive Herzinsuffizienz, HFpEF, Prävalenz, Behandlungsmöglichkeiten, Prognose

## Abstract

Chronic heart failure is one of the most common causes of hospitalization and death in industrialized countries. Demographic changes with an aging population are expected to further increase the prevalence of chronic heart failure. The associated increase in comorbidities in patients with chronic heart failure leads to a less favorable prognosis for survival. A selection of the major comorbidities discussed in this review—along with prevalence, impact on prognosis, treatment approaches, and current study status—include atrial fibrillation, arterial hypertension, coronary artery disease, coronary microvascular dysfunction, renal dysfunction, type 2 diabetes, sleep apnea, reduced lymphatic reserve, and the effects on oxygen utilization and physical activity. The complex clinical picture of heart failure with preserved ejection fraction (HFpEF) remains challenging in the nearly absence of evidence-based therapy. Except for comorbidity-specific guidelines, no HFpEF-specific treatment of comorbidities can be recommended at this time. Optimized care is becoming increasingly relevant to reducing hospitalizations through a seamless inpatient and outpatient care structure. Current treatment is focused on symptom relief and management of associated comorbidities. Therefore, prevention through early minimization of risk factors currently remains the best approach.

## Abstract (de)

Eine der häufigsten Todesursachen in den Industrieländern ist die chronische Herzinsuffizienz. Patienten mit dem Krankheitsbild einer chronischen Herzinsuffizienz gehören zu der Gruppe Patienten, die am häufigsten in die Krankenhäuser eingewiesen wird. Durch den demografischen Wandel mit zunehmender Alterung der Bevölkerung ist ein weiterer Anstieg der Prävalenz der chronischen Herzinsuffizienz zu erwarten. Der damit verbundene Anstieg der Komorbiditäten bei chronischen Herzinsuffizienzpatienten führt zu einer ungünstigeren Überlebensprognose. Eine Auswahl der wichtigsten Komorbiditäten, die in dieser Übersicht mit Prävalenz, Prognose, Behandlungsansätzen und aktuellem Studienstand erörtert werden, umfasst: Vorhofflimmern, arterielle Hypertonie, koronare Herzerkrankung, koronare mikrovaskuläre Dysfunktion, Nierenfunktionsstörung, Typ-2-Diabetes, Schlafapnoe, lymphatische Reserve sowie Auswirkungen auf die Sauerstoffverwertung und körperliche Aktivität. Das komplexe Krankheitsbild der Herzinsuffizienz mit erhaltener Ejektionsfraktion (HFpEF) stellt angesichts einer nahezu fehlenden evidenzbasierten Therapie weiterhin eine Herausforderung dar. Eine optimierte Versorgung wird immer wichtiger, um Krankenhausaufenthalte durch eine nahtlose stationäre und ambulante Versorgungsstruktur zu reduzieren. Die derzeitige Behandlung ist auf die Linderung der Symptome und die Behandlung der damit verbundenen Komorbiditäten ausgerichtet. Daher bleibt aktuell die Prävention durch die frühzeitige Minimierung von Risikofaktoren der beste Ansatz.

Chronic heart failure is one of the most common causes of hospitalization and death in industrialized countries [5, 11]. In the Western world, the prevalence of heart failure is approximately 1–2%, and it increases steadily with age. The incidence is less than 1% in those under 55 years of age and reaches approximately 10% in those older than 70 [25]. According to the terminology given in the European Society of Cardiology (ESC) guidelines, patients with heart failure are divided into three different groups. A distinction is made between patients with normal ejection fraction (HFpEF: left ventricular ejection fraction [LVEF] ≥ 50%) and patients with reduced ejection fraction (HFrEF: LVEF < 40%). The group of patients with LVEF in the range of 40–49% represents a “gray area” and is defined as heart failure with mildly reduced ejection fraction (HFmrEF). Epidemiologic data from the Framingham Study, an international cohort study, shows an increase in the prevalence of HFpEF over the past three decades relative to the overall prevalence of heart failure (from 41% to 56%) and, conversely, a decrease in the prevalence of HFrEF (from 44% to 31%) and HFmrEF (from 15% to 13%; [43]).

Demographic changes with an aging population are expected to further increase the prevalence of chronic heart failure. The associated increase in comorbidities in patients with chronic heart failure leads to a less favorable prognosis for survival, as shown in Fig. 1 [17, 41].

**Fig. 1:**
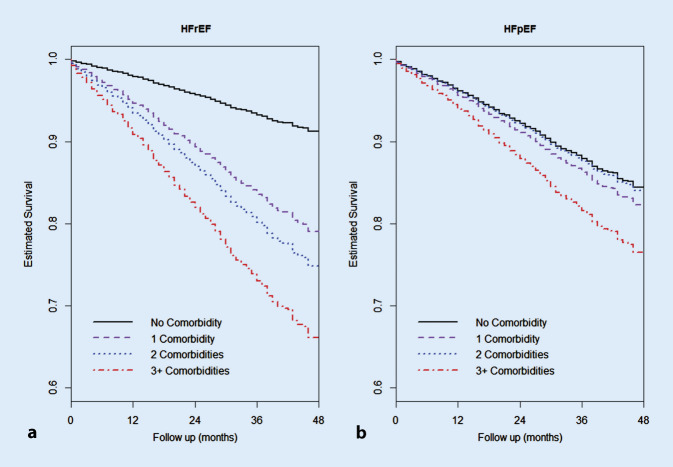
Survival curve according to Cox model of the frequency distribution of patients with comorbidities (0, 1, 2, ≥ 3 comorbidities) with heart failure with preserved ejection fraction (*HFpEF*, **b**) and heart failure with reduced ejection fraction (*HFrEF*, **a**). With permission from [17]

The frequency distribution of patients with comorbidities with HFpEF compared with HFrEF differentiated by men and women is shown in Fig. 2. The graph shows that HFpEF patients have a higher number of concomitant diseases—four on average—than do patients with HFrEF in both sexes [12].

**Fig. 2:**
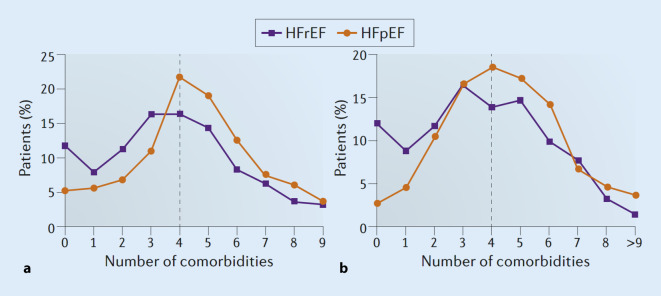
Frequency distribution of comorbidities in women (**a**) and men (**b**) with heart failure with preserved ejection fraction (*HFpEF*, *orange*) and heart failure with reduced ejection fraction (*HFrEF*, *blue*). With permission from [12]

## Comorbidities: prevalence, impact on prognosis, treatment options

Since no HFpEF-specific treatment methods for comorbidities currently exist, treatment recommendations based on comorbidity-specific guidelines are recommended by current guidelines [24].

### Atrial fibrillation

Atrial fibrillation (AF) is the most common sustained cardiac arrhythmia. The estimated prevalence of AF in adults is between 2% and 4% worldwide [15]. If both heart failure and AF coexist, the risk for worse outcomes is not only the summation of each individual disease but it increases exponentially, with a major increase in hospitalizations and a two- to three-fold higher mortality [15, 18].

According to epidemiological studies, there is a substantial association between AF and HFpEF: AF is one of the most common precursors and predictors of the development of HFpEF. Conversely, if the arrhythmia is not already present, most people with HFpEF are destined to develop it [48]. Both conditions are associated with a progressive left atrial myopathy driven by the presence of common cardiovascular risk factors [20]. The coexistence of AF and HFpEF is often underestimated in clinical practice, presumably because unrecognized AF occurs years before patients receive a diagnosis, and patients suffer from exertional dyspnea before physicians detect the presence of heart failure. The diagnosis of HFpEF on the basis of natriuretic peptides is very limited or even impossible in AF patients with suspected HFpEF [44].

Studies showing an exceptionally high prevalence of HFpEF in patients with AF, exertional dyspnea, and a normal ejection fraction support these associations. Reddy et al. showed that when patients with exertional dyspnea underwent exercise right heart catheterization, up to 64% suffered from occult HFpEF (pulmonary capillary wedge pressure of ≥ 25 mm Hg on exertion; [31]). To determine the impact of AF ablation on these patients the STALL AF-HFpEF trial (*St*udy Using Inv*a*sive Hemodynamic Measurements Fo*ll*owing Catheter Ablation for AF and Early HFpEF) evaluated 54 patients referred for catheter ablation for AF (with or without dyspnea on exertion; [39]).

Overall, 65% of these patients met diagnostic criteria for HFpEF in invasive measurements (pulmonary capillary wedge pressure of ≥ 25 mm Hg during exercise) and 92% of those with persistent AF fulfilled diagnostic criteria for HFpEF. After a follow-up of 12 months, nine patients (45%) who underwent ablation showed significant improvement in pulmonary wedge pressure and quality of life. Both studies showed a high rate of undetected HFpEF as an exceptionally common disorder in patients with AF who present with exertional dyspnea.

Treatment strategies for AF mainly differentiate between rate or rhythm control. Pharmacological rate control in HFpEF patients is difficult, treatment options are limited, and many antiarrhythmic drugs are contraindicated or poorly tolerated due to extracardiac side effects and high discontinuation rate (e.g. amiodarone).

The AFFIRM (Atrial Fibrillation Follow-Up Investigation of Rhythm Management) trial showed that if antiarrhythmic drugs (beta-blockers, calcium channel blockers, digoxin, or a combination of these medications) only were used, rate control equaled rhythm control in longer-term follow-up regarding outcomes such as mortality and stroke in patients with HFrEF. Furthermore, the stroke rate in the rhythm control arm was very high, mostly due to (inadequate) termination of oral anticoagulation [10]. These results sparked interest in rhythm control by AF ablation and prompted investigators to study the safety and practicality of AF ablation in heart failure patients. The focus of AF rhythm control therapy shifted toward catheter ablation. Several trials showed that catheter ablation improves clinical outcomes in AF patients with HFrEF [19, 22, 45]. However, the role of catheter ablation in HFpEF is less clear and data on the role of atrial fibrillation ablation in HFpEF are currently sparse.

The CABANA trial (Catheter Ablation vs. Antiarrhythmic Drug Therapy for Atrial Fibrillation) randomized patients with AF to either pulmonary vein isolation or antiarrhythmic drugs (rate or rhythm control). Only 9.3% of the patients had an LVEF < 40% and the median LVEF was 55%, implying a population with HFpEF rather than a population with HFrEF. Pulmonary vein isolation was not superior to drug therapy for cardiovascular outcomes at 5 years [27].

Yamauchi et al. showed in a large observational study of nearly 300 randomized patients with HFpEF, that these patients had similar ablation outcomes to HFrEF patients and patients without heart failure, although a majority of patients suffered from recent-onset or paroxysmal AF [46].

Similar to this observational study, a meta-analysis published in 2021 showed no significant differences in rates of AF recurrence 1 year after catheter ablation as well as improvements in New York Heart Association functional class and symptoms in AF-dedicated quality-of-life scores between patients with HFpEF and those with HFrEF [1].

The current ESC guidelines on heart failure treatment recommend the prudent use of AF catheter ablation (class IIa), and there is no difference between the recommendations for patients with HFpEF and HFrEF [23].

Further randomized clinical trials evaluating the clinical outcomes of catheter ablation and rhythm control therapy in AF patients with HFpEF are needed. In any such trial, a sham-controlled comparator should be used.

### Arterial hypertension

The most common comorbidity in HFpEF patients is hypertension, which can be diagnosed in approximately 75% of HFpEF patients. Several studies investigated the impact of blood pressure control on outcomes in hypertensive patients with HFpEF. Tsujimoto et al. analyzed data from the TOPCAT study of 3417 HFpEF patients. Low systolic blood pressure in HFpEF patients was found to be an independent predictor of short- and long-term mortality in this population. In patients with mild hypertension, systolic blood pressure between 120 and 130 mm Hg and diastolic blood pressure between 70 and 80 mm Hg were associated with the lowest all-cause mortality [42]. Arterial hypertension affects myocardial remodeling and dysfunction in HFpEF patients through myocardial overload and systemic inflammation [6, 32]. Furthermore, hypertension causes activation of the renin–angiotensin–aldosterone system and sympathetic nervous system with increased catecholamine release, which leads to down-regulation of beta receptors, an increase in afterload, and thus further worsening of heart failure. Diuretics, spironolactone, angiotensin-converting enzyme inhibitors, and angiotensin II receptor blockers, based on currently available data, are therefore the first choice, along with nonpharmacological agents, to control blood pressure as the main prevention and treatment strategy in HFpEF patients [28]. In a 2018 meta-analysis of 11 large, randomized trials of beta-blocker therapy across the heart failure spectrum, there were no benefits in reducing cardiovascular morbidity and mortality in a small subcohort with HFpEF and sinus rhythm [8]. In the ELANDD trial, nebivolol failed to positively affect heart failure symptoms in HFpEF. It was shown that peak oxygen uptake (peak VO_2_) decreased slightly in the nebivolol group, and increased in the placebo group, without reaching statistical significance. Resting and peak blood pressure, as well as systolic blood pressure, decreased significantly from baseline in the nebivolol group, without a change in the placebo arm [9]. Therefore, beta-blocker therapy cannot be recommended in HFpEF patients unless there are other reasons for this therapy, such as coronary artery disease.

### Coronary artery disease

Coronary artery disease (CAD) is a common concomitant disease, detectable in more than 50% of HFpEF patients [26]. When considering the prognosis of CAD, significant differences are seen in HFpEF patients compared with HFrEF patients. The risk of cardiovascular death, as well as the incidence of sudden death, is significantly higher in HFpEF patients with CAD compared with HFrEF patients with CAD [34]. Stenosing coronary arteries cause a reduction in coronary flow reserve as well as oxygen supply in the myocardium, leading to a decrease in diastolic functional reserve. Furthermore, structural remodeling with compensatory hypertrophy, scarring, and impaired relaxation occurs as a result of myocardial infarction. Observational data from HFpEF patients with CAD suggest that complete revascularization is associated with better preservation of left ventricular systolic function and improved prognosis [16]. International guidelines consistently recommend that patients with chronic heart failure and CAD be treated analogously to patients with CAD without heart failure. The treatment and prevention of ischemia and coronary events should be the primary focus [30, 47].

### Coronary microvascular dysfunction

**Fig. 3:**
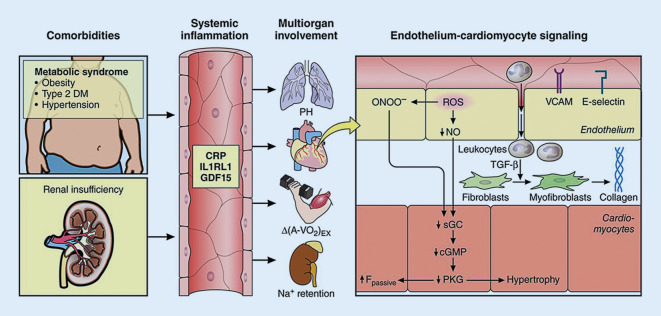
Systemic and myocardial signaling in heart failure with preserved ejection fraction. *cGMP* cyclic guanosine monophosphate, *CRP* C-reactive protein, *DM* diabetes mellitus, *GDF15* growth differentiation factor-15, *IL1RL1* interleukin 1 receptor-like 1, *NO* nitric oxide, *ONOO*^−^ peroxynitrite, *PKG* protein kinase G, *ROS* reactive oxygen species, *sGC* soluble guanylate cyclase, *TGF* transforming growth factor, *VCAM* vascular cellular adhesion molecule. With permission from [35]

Coronary microvascular dysfunction (CMD) is discussed as a novel mechanism underlying the pathogenesis of HFpEF (Fig. 3; [35]). It has been hypothesized that comorbidities associated with HFpEF lead to systemic as well as to coronary endothelial inflammation and CMD, which reduce endothelial nitric oxide bioavailability and cyclic guanosine monophosphate production by adjacent cardiomyocytes. This process leads to downstream titin hypophosphorylation and increased cardiomyocyte stiffness and hypertrophy, myofibroblast activation, and interstitial fibrosis. Both cardiomyocyte and extracellular mechanisms lead to increased left ventricular diastolic stiffening, a well-known feature of HFpEF syndrome [36]. The role of CMD is not yet fully explained but may contribute to the development of new therapeutic strategies for patients with HFpEF.

### Renal dysfunction

Renal dysfunction is also a common comorbidity in HFpEF patients. Over 20–30% of patients with HFpEF have chronic kidney failure. Heart failure and renal dysfunction influence each other, with cardiovascular risk and mortality increasing with decreasing renal function [29, 40]. Renal blood flow and sodium excretion are reduced by increased central venous pressure resulting from pulmonary hypertension and right ventricular dysfunction. Renal dysfunction, in turn, promotes HFpEF by worsening systemic inflammation and endothelial dysfunction, due in part to renal mediators such as high levels of fibroblast growth factors or uremic toxins [35]. The concomitant cardiac and renal insufficiency in patients poses several clinical challenges, as many established heart failure medications can cause worsening renal function or are contraindicated in the presence of renal insufficiency. Clinical experience shows that there are often mild fluctuations in renal function in patients with chronic heart failure, but an increase in serum creatinine above 30% of the baseline is usually not exceeded. In acute worsening of renal function, dose reduction or discontinuation of renin–angiotensin–aldosterone system inhibitors and diuretics in the presence of dehydration is recommended in current guidelines. Close monitoring of electrolyte balance and renal function is required. Basic drug therapy with angiotensin-converting enzyme inhibitors or angiotensin receptor blockers, beta receptor blockers, and mineralocorticoid receptor antagonist is recommended, taking contraindications into account and with careful titration or adjustment of the dosage.

### Type 2 diabetes

Type 2 diabetes (T2D) is a high-risk factor in patients with HFpEF and plays a significant role in diastolic dysfunction. Approximately one third of HFpEF patients have concomitant diabetes mellitus [29]. Furthermore, T2D has been described as a comorbidity with a high risk of mortality and hospitalization [21]. Diabetes mellitus causes functional, morphologic, and biochemical changes in the myocardium that can lead to diastolic dysfunction and heart failure independent of other cardiovascular risk factors [14]. Intensified glycemic control, as shown in numerous studies, did not have a positive effect on cardiovascular mortality or hospitalization for heart failure, but instead increased susceptibility to hypoglycemia [7]. Current guidelines for the treatment of T2D recommend HbA1c levels in the range of 7%, and the treatment goal regarding HbA1c levels should be adjusted considering certain factors, such as age, comorbidities, hypoglycemia risk, and diabetes duration. Sodium-dependent glucose co-transporter 2 inhibitors are currently profiled as a therapeutic option to improve prognosis in heart failure patients with and without T2D.

The EMPEROR-Preserved study, a multicenter, double-blind, phase III trial enrolled 5988 symptomatic HFpEF patients (LVEF over 40%), both with and without T2D, across 23 countries. Participants were randomized in a 1:1 ratio to receive either 10 mg empagliflozin or placebo once daily, in addition to standard-of-care therapies. Over a median follow-up of 26.2 months, 13.8% of empagliflozin-treated patients and 17.1% of placebo-treated patients experienced a primary outcome event, equating to a hazard ratio of 0.79 (*p* < 0.001). This effect was observed across subgroups, including patients with and without T2D, as well as patients with an LVEF of less than 50%, 50–60%, or 60% and more. The trial results confirm that empagliflozin reduced the risk of a composite of cardiovascular death or hospitalization for heart failure in both diabetic and non-diabetic patients with HFpEF compared to placebo [2]. Another multicenter, international, double-blind, phase III trial, the DELIVER study, evaluating the efficacy of dapagliflozin in HFpEF patients compared to placebo, has already started. The first results from the DELIVER trial showed that dapagliflozin reached a statistically significant reduction in the primary composite endpoint of cardiovascular death or worsening heart failure. The full results are currently expected in the next few months [4, 37, 38].

### Sleep apnea

Another very common comorbidity of heart failure is sleep apnea, which occurs in approximately 48% of HFpEF patients. A distinction must be made between obstructive (OSA) and central sleep apnea (CSA). Both OSA and CSA are associated with increased mortality in HFpEF patients [3]. Therefore, heart failure patients should always be monitored for corresponding symptoms such as daytime sleepiness, nocturnal breathing pauses, tendency to fall asleep, etc. In the case of abnormalities, further diagnostics should be initiated. Differentiation between OSA and CSA using polysomnography is important for appropriate therapy. In HFpEF patients, the proportion of patients with OSA predominates. A central role in OSA is the treatment of known triggering factors, such as obesity or excessive alcohol consumption. Furthermore, discontinuation or reduction of triggering medications such as opiates should be discussed. Often, CSA is caused by heart failure as the underlying disease and can be improved by optimal heart failure therapy.

### Skeletal muscle, oxygen utilization, and physical activity

Several studies indicate that peak VO_2_ is significantly reduced in HFpEF patients. These patients exhibit abnormalities in skeletal muscle mass, composition, capillary density, and oxidative metabolism. Haykowsky et al. showed that elderly HFpEF patients have significantly reduced lean body mass and lean leg mass on a percentage basis compared with age-matched healthy patients. When peak VO_2_ was indexed to total lean body mass or lean leg mass, peak VO_2_ remained significantly reduced [13]. Thus, HFpEF patients have abnormal oxygen utilization that is independent of, and in addition to, their reduced muscle mass. Furthermore, HFpEF patients showed abnormal skeletal muscle composition with infiltration of adipose tissue, which is directly related to their reduced maximal oxygen uptake.

Endurance training leads to improved exercise capacity in HFpEF patients primarily by improving mitochondrial skeletal muscle mass and function. On the other hand, high-intensity and strength training have not yet been systematically studied.

### Reduced lymphatic reserve

**Fig. 4:**
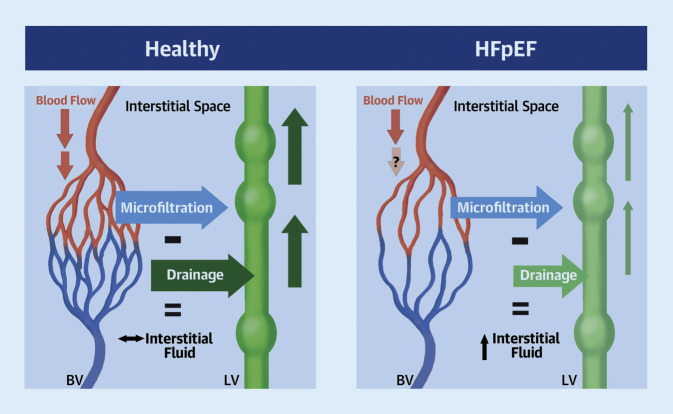
Microvascular fluid dynamics and reduced lymphatic reserve in heart failure with preserved ejection fraction (*HFpEF*). *BV* blood vasculature, *LV* lymphatic vasculature. With permission from [33] under the terms of the Creative Commons CC-BY license

Microvascular dysfunction plays an important role in the pathogenesis of HFpEF (Fig. 4). In patients with HFpEF, peripheral lymphatics show structural and molecular alterations. In a 2020 study by Rossitto et al. with 32 patients, these morphological and functional alterations in the lymphatic vasculature were demonstrated in HFpEF patients, leading to decreased clearance of extravascular fluid and thus higher interstitial fluid accumulation. A better understanding of these mechanisms may provide a new pharmacological target for HFpEF treatment [33].

### Further comorbidities

Other comorbidities such as anemia, depression, obesity, hyperlipidemia, chronic obstructive pulmonary disease, sarcopenia, and pulmonary hypertension should be mentioned in this context. These are independent risk factors for the development of heart failure and are frequently found in heart failure patients.

## Conclusion

The complex clinical picture of heart failure with preserved ejection fraction (HFpEF) remains challenging in the absence of evidence-based therapy. Except for comorbidity-specific guidelines, no HFpEF-specific treatment of comorbidities can be recommended at this time. Optimized care, especially for heart failure patients, is becoming increasingly relevant to reducing hospitalizations through a seamless inpatient and outpatient care structure. Current treatment is focused on symptom relief and management of associated comorbidities. Therefore, prevention through early minimization of risk factors currently remains the best approach. Further studies and new scientific knowledge are needed that will contribute to a better understanding of this complex syndrome.

## Declarations

For this article no studies with human participants or animals were performed by any of the authors. All studies mentioned were in accordance with the ethical standards indicated in each case.
